# Human α4β2 Nicotinic Acetylcholine Receptor as a Novel Target of Oligomeric α-Synuclein

**DOI:** 10.1371/journal.pone.0055886

**Published:** 2013-02-20

**Authors:** Qiang Liu, Sharareh Emadi, Jian-Xin Shen, Michael R. Sierks, Jie Wu

**Affiliations:** 1 Divisions of Neurology, Barrow Neurological Institute, St. Joseph’s Hospital and Medical Center, Phoenix, Arizona, United States of America; 2 Department of Chemical Engineering, Arizona State University, Tempe, Arizona, United States of America; 3 Department of Physiology, Shantou University of Medical College, Shantou, People’s Republic of China; 4 Department of Basic Medical Sciences, University of Arizona College of Medicine Phoenix, Arizona, United States of America; University of Melbourne, Australia

## Abstract

Cigarette smoking is associated with a decreased incidence of Parkinson disease (PD) through unknown mechanisms. Interestingly, a decrease in the numbers of α4β2 nicotinic acetylcholine receptors (α4β2-nAChRs) in PD patients suggests an α4β2-nAChR-mediated cholinergic deficit in PD. Although oligomeric forms of α-synuclein have been recognized to be toxic and involved in the pathogenesis of PD, their direct effects on nAChR-mediated cholinergic signaling remains undefined. Here, we report for the first time that oligomeric α-synuclein selectively inhibits human α4β2-nAChR-mediated currents in a dose-dependent, non-competitive and use-independent manner. We show that pre-loading cells with guanyl-5′-yl thiophosphate fails to prevent this inhibition, suggesting that the α-synuclein-induced inhibition of α4β2-nAChR function is not mediated by nAChR internalization. By using a pharmacological approach and cultures expressing transfected human nAChRs, we have shown a clear effect of oligomeric α-synuclein on α4β2-nAChRs, but not on α4β4- or α7-nAChRs, suggesting nAChR subunit selectivity of oligomeric α-synuclein-induced inhibition. In addition, by combining the size exclusion chromatography and atomic force microscopy (AFM) analyses, we find that only large (>4 nm) oligomeric α-synuclein aggregates (but not monomeric, small oligomeric or fibrillar α-synuclein aggregates) exhibit the inhibitory effect on human α4β2-nAChRs. Collectively, we have provided direct evidence that α4β2-nAChR is a sensitive target to mediate oligomeric α-synuclein-induced modulation of cholinergic signaling, and our data imply that therapeutic strategies targeted toward α4β2-nAChRs may have potential for developing new treatments for PD.

## Introduction

Parkinson disease (PD) is one of the most common neurodegenerative disorders affecting more than half a million people in the United States, with annual costs estimated at 10 billion dollars [1]. The neuropathological hallmarks of PD are progressive loss of dopaminergic neurons in the substantia nigra pars compacta (SNc) and microscopic proteinaceous inclusions, composed mainly of aggregated fibrillar α-synuclein in neurons and glia [2], [3]. α-Synuclein, an abundant presynaptic protein in the central nervous system (CNS), consists of a 140 amino-acid sequence that is highly homologous across human, rat and mouse [3]. Although the precise mechanisms of PD pathogenesis are only partially understood, it is now widely accepted that the accumulation and aggregation of α-synuclein plays a crucial role in the pathogenesis of PD. α-Synuclein has been tightly linked to PD [4], [5] and other related neurodegenerative disorders such as multiple systems atrophy (MSA), Hallervorden-Spatz disease, neurodegeneration with brain iron accumulation type-1, and Niemann-Pick Type C Disease [6], [7]. Additionally, over expression of α-synuclein in transgenic models has been shown to induce the formation of PD-like pathological phenotypes and behavior, despite absence of neuronal loss in the CNS [8], [9]. α-Synuclein is considered a cytosolic protein, and consequently its pathogenic effect was assumed limited to the cytoplasm of single cells [10]. However, recent studies have suggested that α-synuclein also has extracellular pathogenic effects [11], [12], [13], [14]. α-Synuclein has been detected in blood plasma and cerebrospinal fluid in both monomeric and oligomeric forms [11], [12], [13], [14], and the presence of significantly elevated levels of oligomeric species of α-synuclein has been reported in plasma and cerebrospinal fluid samples from patients with PD [12]. Furthermore, various studies have shown that the extracellular addition of aggregated α-synuclein to culture medium is cytotoxic [15],[16],[17],[18],[19],[20],[21].

It has been reported that cigarette smoking is associated with a lower incidence of PD, attributed to a neuroprotective effect of nicotine through the activation of nicotinic acetylcholine receptors (nAChRs) [22], [23], [24], [25]. Previous studies indicate extensive expression and function of the nAChRs in midbrain dopaminergic neurons [26], [27], [28], [29], and a decrease of nAChRs, especially α4β2-nAChR and α6β2-nAChR binding sites has been observed in PD patients’ brain [30], [31], [32], [33]. Moreover, recent evidence suggests possible roles for nAChRs as potential targets for α-synuclein-induced neurotoxicity resulting in cholinergic hypofunction and neuronal degeneration in basal ganglia [26], [27], [33]. Collectively, these findings point to a possible abnormality of nAChRs assembly and function in PD and highlight nAChRs as potential targets to prevent or treat PD. However, the link between nAChRs and α-synuclein, the major pathogen in PD, remains obscure and undefined, and there is little evidence indicating whether α-synuclein, particularly different forms of α-synuclein, can directly affect nAChRs function.

Considering the significant loss of nAChRs in PD brain with α-synuclein over expression, the neurotoxicity of α-synuclein to SNc dopaminergic neurons, the extensive distribution of cholinergic innervations and their receptors in SNc dopaminergic neurons, and the neuroprotective effects provided by nAChR activation [27], [28], [29], it is reasonable to hypothesize that α-synuclein might perturb cholinergic signaling by impairing nAChRs function. To test this hypothesis, in the present study we employed patch-clamp techniques combined with size exclusion chromatography and atomic force microcopy (AFM) analyses to examine and elucidate the acute effects of specific forms of α-synuclein on the function of human α4β2-nAChRs heterologously expressed in the human SH-EP1 cell line.

## Methods

### Heterologously Expressed Human α4β2-, α7- and α4β4 nAChRs in SH-EP1 Cells

Human α4, α7, β2, and β4 subunits were subcloned into pcDNA3.1-zeocin and pcDNA3.1-hygromycin vectors, and transfected using established techniques [34], [35], [36] into native nAChR-null SH-EP1 cells [37] to create the SH-EP1-hα4β2 cell line. For this and all other methods, manipulations were conducted at room temperature (23±1°C) unless otherwise noted. Briefly, 3 million SH-EP1 cells in 0.5 ml of 20 mm HEPES, 87 mm NaCl, 5 mm KCl, 0.7 mm NaHPO_4_, 6 mm dextrose, pH 7.05, in an electroporation cuvette were mixed with α7, α4+β2, or α4+β4 subunit cDNA constructs. Samples were subjected to electroporation (Bio-Rad Gene Pulsar model 1652076) at 960 microfarads and 200 volts. After electroporation, cells were suspended to 5 ml in complete medium [38], and 1-ml aliquots were added to 12-ml aliquots of medium in each of five 100-mm dishes before returning the cells to an incubator at 37°C. 48 h later, positive selection of incubated cells was initiated by supplementing the medium with 0.25 mg/ml zeocin (Invitrogen, NY) and 0.4 mg/ml hygromycin (Calbiochem, CA). Colonies of surviving cells were selected by ring cloning and expanded before being screened for radioligand binding and functional evidence for nAChR expression, which led to selection of the clone designated as the SH-EP1-human nAChR cell line. Cells were maintained at low passage numbers in medium with 0.25 mg/ml zeocin and 0.4 mg/ml hygromycin to ensure stable expression of phenotype and passaged once weekly by splitting just-confluent cultures 1/10 to maintain cells in proliferative growth.

### Patch-clamp Whole Cell Recordings

Conventional whole cell current recording, coupled with techniques for fast application and removal of drugs (two-barrel tubes, Warner Instrument), was applied in this study as previously described [39], [40], [41]. Briefly, cells plated on polylysine-coated 35-mm culture dishes were placed on the stage of an inverted microscope (Olympus iX7, Lake Success, NY) and continuously superfused with standard external solution (2 ml/min). Glass microelectrodes (3–5 MΩ resistance between pipette and extracellular solutions) were used to form tight seals (>2 GΩ) on the cell surface until suction was applied to convert to conventional whole cell recording. Cells were then voltage-clamped at a holding potential of −60 mV, and ion currents in response to application of ligands were measured (Axon Instruments 200 B amplifier, Molecular Devices, Sunnyvale, CA), typically using data filtered at 2 kHz, acquired at 10 kHz, displayed and digitized on-line (Axon Instruments Digidata 1322 series A/D board), and stored on hard media for subsequent off-line analysis. Both pipette and whole cell current capacitance were minimized, and the series resistance was routinely compensated to 80%. Before series resistance compensation, whole cell access resistance less than 20 MΩ was accepted. Data acquisition and analyses were done using Pclamp9.2 (Axon Instruments, Molecular Devices, Sunnyvale, CA), and results were plotted using Origin 5.0 (Microcal, North Hampton, MA). The drugs used in this study are nicotine bitartrate, acetylcholine, choline and GDP-β-S, which were purchased from Sigma Aldrich (St. Louis, MO). Amyloid peptide 1–42 was purchased from r-Peptide (Bogart, GA), and α-synuclein was provided by Dr. Sierks.

### Production and Purification of α-synuclein

α-Synuclein was prepared and purified as previously described [19], [42]. Briefly, α-synuclein plasmid was transformed into BL-21 competent cells, plated onto LB-agar plates (supplemented with 100 µg/ml ampicillin), and grown overnight at 37°C. Single colonies of BL21 (DE3) were grown and purified essentially as described [42]. α-Synuclein was lyophilized and stored at –80°C.

### Production and Determination of Oligomeric and Fibrillar α-synuclein

The lyophilized α-synuclein stock was dissolved in buffer (25 mM Tris-HCl and 150 mM NaCl, pH 7.4) to a concentration of 70 µM. Oligomeric aggregates of α-synuclein were obtained by incubating at 37 °C for 7–10 days without shaking. Fibrils were obtained upon longer incubation up to 35 days. Atomic force microscopy (AFM) was used to determine the morphologies of the synthetically prepared α-synuclein aggregates. Topographic AFM images were obtained in air at room temperature using a Tapping Mode AFM with a Nanoscope IIIa controller (Veeco, Santa Barbara, CA). Images were acquired using oxide sharpened Si_3_N_4_ AFM tips (k = 40 N/m, f_o_ ∼300 kHz) (Model: OTESPA, Veeco, Santa Barbara, CA) at scan rates of 2–3 Hz and at scan resolution of 512 samples per line. Images were subjected to 2^nd^ order polynomial flattening as needed to reduce the effects of image bowing and tilt. AFM images were analyzed with the Scanning Probe Imaging Processor (SPIP) software (Image Metrology, www.imagemet.com) to generate height distribution histograms for each sample.

### Data Analysis and Statistics

nAChR acute desensitization (the decline in inward current amplitude over the course of agonist application) was analyzed for decay half-time (τ; τ = 0.693/k for decay rate constant *k*), peak current (*I_p_*), and steady-state current (*I_s_*), by fitting to the mono- (or double-) exponential expression *I* = [(*I_p_*−*I_s_*) *e* ^−kt^]+*I_s_* (or *I* = [(*I_p_*−*I_i_*) *e* ^−k1t^]+[(*I_i_*−*I_s_*) *e* ^−k2t^]+*I_s_*, where *I_i_* is the intermediate level of current and *k*_1_ and *k*_2_ are rate constants from the two separate decay processes). The statistical significance of the comparison between two groups of matched data sets was assessed as *p*<0.05 using two-tailed Student’s *t*-test. All values are expressed as mean ± SEM. For statistical analysis of data from multiple groups of data, one-way or multivariate ANOVA followed by appropriate test were applied. All experiments were performed at room temperature (23±1°C). Dose-response profiles were fit to the Hill equation and analyzed using Prizm 3.0.

## Results

### Oligomeric α-synuclein Inhibits h*α*4*β*2**-**nAChR-mediated Whole-cell Currents

**Figure 1:**
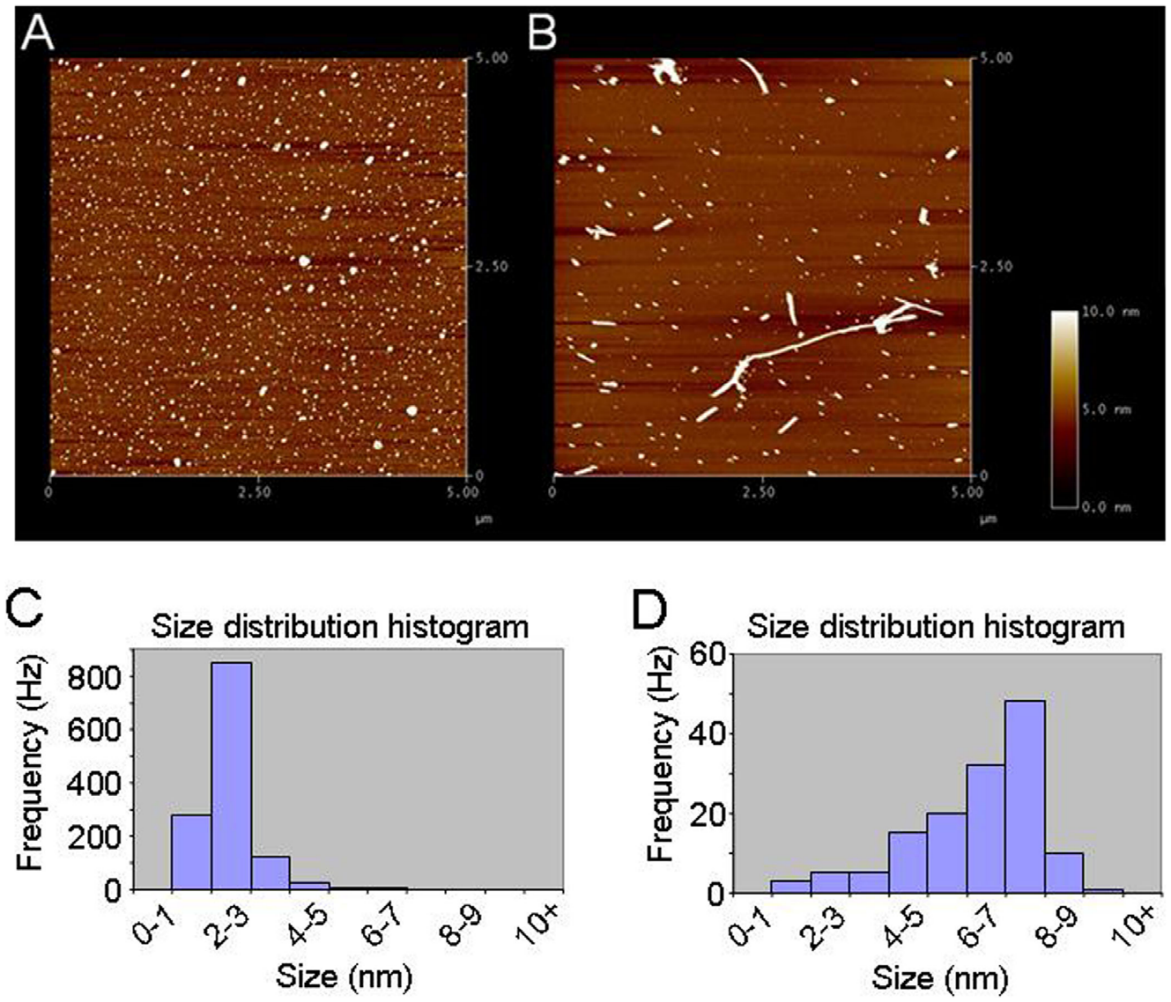
Characterization of α-synuclein oligomeric species by size exclusion chromatography. A . AFM image of α-synuclein pre-incubated at 37°C for 7 days. **B**. Fibrillar aggregates of α-synuclein. **C** and **D.** The size of particles was measured on the AFM height images by using Scanning Probe Image Processor 4.5.5 (Image Metrology A/S, Hoesholm, Denmark). The typical height of α-synuclein oligomers (**C**) is at 2–3 nm, comparatively the height of fibrils (**D**) is at 7–8 nm.

**Figure 2:**
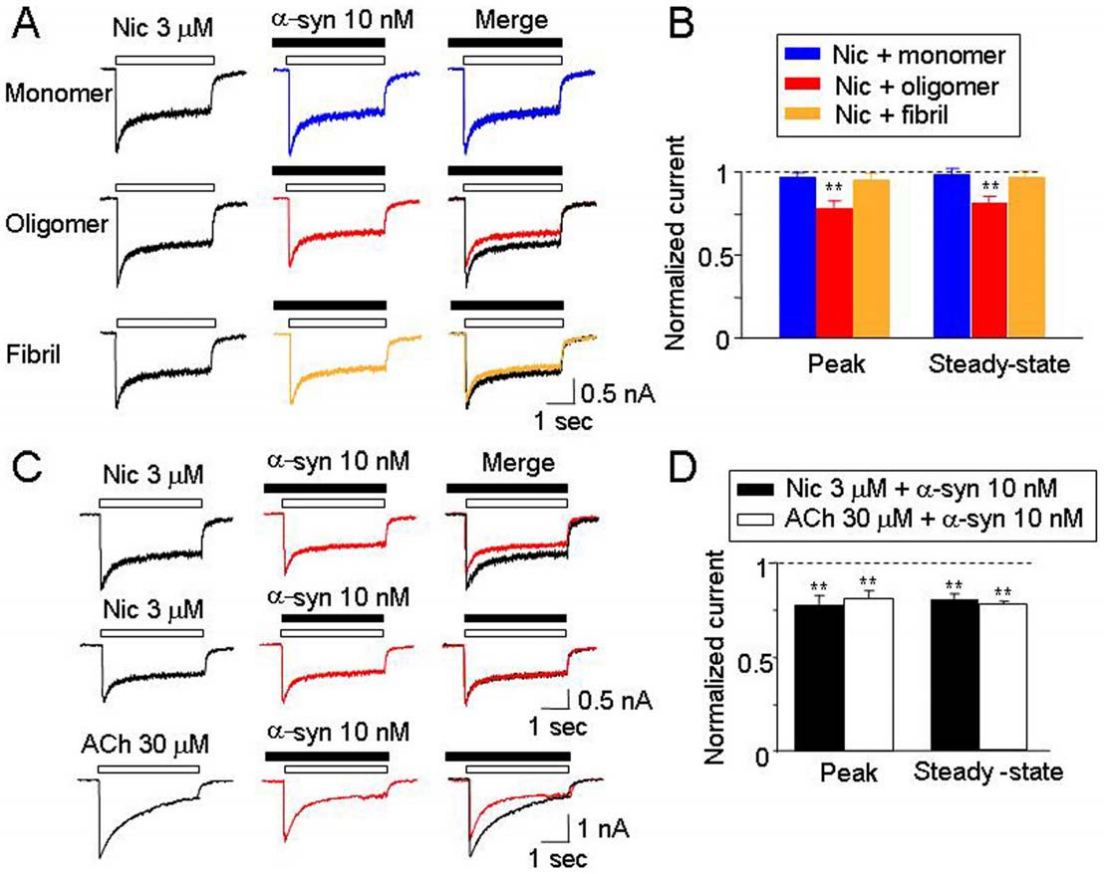
α-Synuclein acutely modulates hα4β2-nAChR-mediated currents. A. Effects of monomeric, oligomeric and fibrillar forms of α-synuclein on hα4β2-nAChR- mediated whole-cell currents induced by nicotine. B. Bar graph summarizes results (peak and steady-state currents) from replicate studies. The horizontal dashed line indicates the control value (normalized as 1.0) for the specified parameters for the nicotinic response (induced by 3 µM nicotine) before α-synuclein treatment. Double asterisk means *p*<0.01. **C**. Comparison of effects of 10 nm α-synuclein (oligomer) on nicotine-(with or without pretreatment of 10 nm α-synuclein) and ACh-induced currents. Whole-cell current response traces induced by nicotine recorded from the same cell without (Black) or with α-synuclein (Red), and currents induced by ACh from another cell without (Black) or with α-synuclein (Red), respectively. **D**. Bar graph summarizes results (peak and steady-state currents) from replicate studies. In all recordings, the cells were held at a holding potential (V_H_) of −60 mV.

Morphologically distinct oligomeric and fibrillar forms of α-synuclein were generated by incubating monomeric α-synuclein for different lengths of time and aggregate morphologies were analyzed by AFM (Fig. 1A and B). Initial experiments were designed to examine the acute effects of different forms of α-synuclein (10 nM monomeric equivalent) on hα4β2-nAChR-mediated currents. Oligomeric but not monomeric or fibrillar forms of α-synuclein inhibited nicotine-induced whole-cell currents (Fig. 2A). Within 2 min of pretreatment, oligomeric α-synuclein inhibited the nicotine-induced peak (reduced to 77.9±4.1% of control values for nicotine, *n* = 8, *p*<0.01, t-test; Fig. 2A and B) and steady-state currents (reduced to 82.8±2.3% of control values, *n* = 8, *p*<0.01, t-test; Fig. 2B). Similarly, oligomeric α-synuclein inhibited the ACh-induced peak (reduced to 81.5±4.2% of control values, *n* = 8, *p*<0.01, t-test) and steady-state whole cell currents (reduced to 78.3±1.9% of control values, n = 8, p<0.01, t-test; Fig. 2C and D). However, without pretreatment, 10 nM oligomeric α-synuclein (co-application with nicotine) did not exhibit significant inhibition of peak current responses to nicotine (Fig. 2C and D; 93.7% ±8.2, n = 8, *p*>0.05, one-way ANOVA). Taken together, these data suggest that oligomeric α-synuclein aggregates acutely inhibit hα4β2-nAChR function.

### α-Synuclein Differentially Inhibits h*α*4*β*2-nAChR-mediated Whole-cell Currents Depending on Aggregate Morphology

To further characterize the specific aggregate morphologies of α-synuclein involved in altering hα4β2-nAChR function, we used size-exclusion chromatography to separate a 7-day pre-aggregated α-synuclein sample into different aggregated species. The 7-day aggregated sample contains high concentrations of different oligomeric α-synuclein species. We obtained three distinct aggregated forms of α-synuclein particles from 7-day oligomeric aggregates and determined the height distribution of these particles by AFM as previously described [10], where the largest aggregate have heights greater than 4 nm, smaller aggregates have heights between 1 and 4 nm and monomeric particles have heights less than 1 nm [10]. Therefore, we can obtain samples predominantly containing large oligomeric (>4 nm), small oligomeric (1–4-nm), or monomeric α-synuclein.

**Figure 3:**
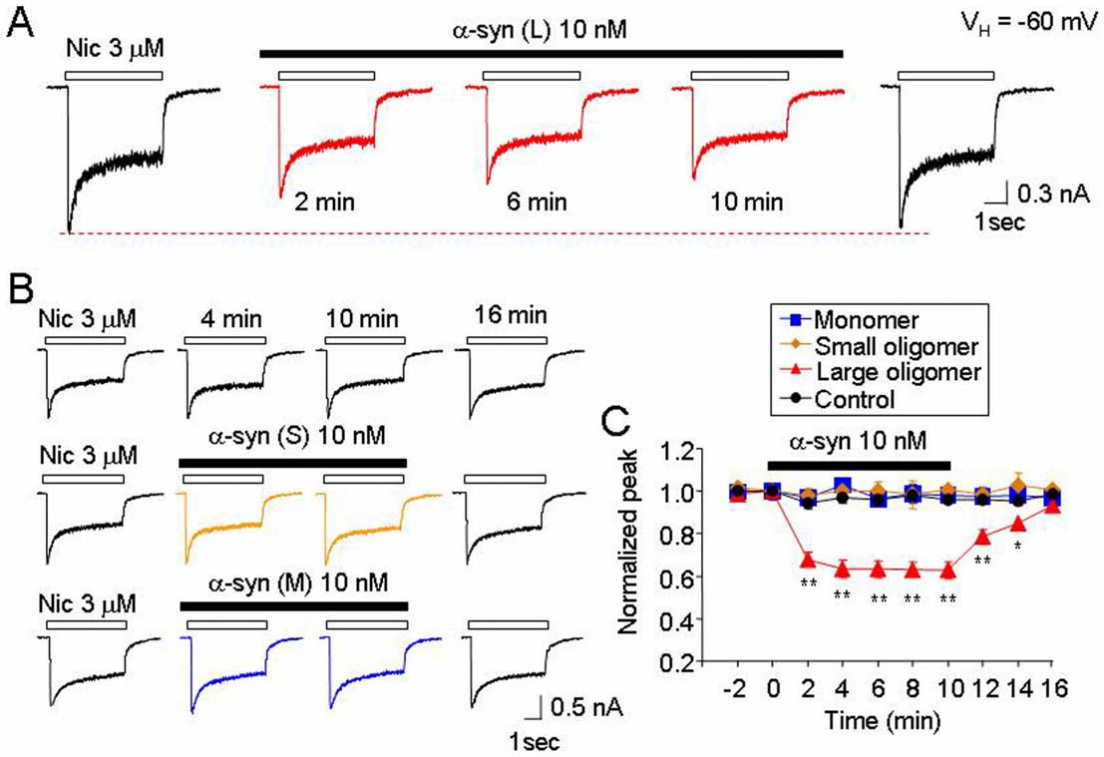
Effects of different morphological sizes of α-synuclein oligomers on hα4β2-nAChR function. A . Typical traces illustrating the effects of 10 nM large oligomeric α-synuclein [α-syn (L), >4 nm] on 3 µM nicotine-induced whole-cell currents recorded from hα4β2-nAChRs expressed in SHEP1 cells. **B**. Representative typical traces of nicotine-induced whole-cell currents in the absence and presence of monomeric or small oligomeric α-synuclein (2–4 nm). **C.** Summary of experimental results from **A** and **B**. Each symbol was averaged from 6 cells tested. The vertical bars indicate Mean ± SE. The single asterisk means *p*<0.05, the double asterisk means *p*<0.01.

We compared the effects of the morphologically distinct oligomeric α-synuclein aggregates on hα4β2-nAChR-mediated whole-cell currents. While the large oligomeric α-synuclein aggregates significantly inhibited h*α*4*β*2-nAChR-mediated whole-cell currents (Fig. 3A), the small aggregates or monomers did not (Fig. 3B). Statistical analysis showed that after 10 min exposure of large oligomeric, small oligomeric or monomeric α-synuclein, the nicotinic responses (normalized peak amplitude) were reduced to 63.5±3.9% (n = 6, p<0.05 or p<0.01, multi-variate ANOVA), 93.2±5.6% (n = 6, p>0.05, multi-variate ANOVA) or 92.5±6.3% (n = 6, *p*>0.05, multi-variate ANOVA), respectively. These findings suggest that the inhibition of h*α*4*β*2-nAChR-mediated whole-cell currents by oligomeric α-synuclein is mediated by large size oligomeric α-synuclein.

### α-Synuclein Inhibits Human α4β2-nAChR-mediated Currents in Non-competitive

**Figure 4:**
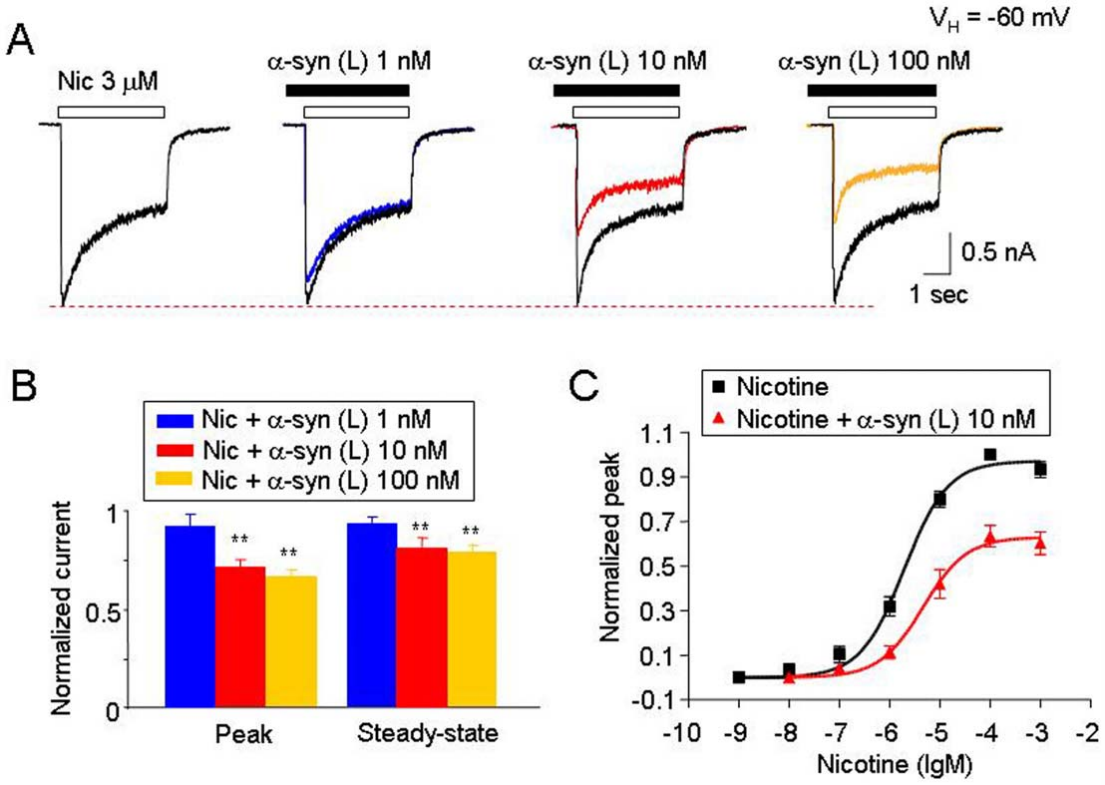
α-Synuclein inhibits hα4β2-nAChR-mediated currents in a dose-dependent and non-competitive manner. A . Representative typical traces (recorded from the same cell) of nicotine-induced whole-cell currents in the presence of different concentrations of large oligomeric α-synuclein. **B**. Summary of pool results for the effects of different concentrations of α-synuclein. **C**. Effects of α-synuclein (10 nM, large oligomeric) on the concentration-response curves of hα4β2-nAChR-mediated whole-cell currents. Functional fit to the logistic equation indicates that in the presence of α-synuclein, the maximal current response in the agonist dose-response profile was significantly reduced without change of apparent EC_50_ for agonist (nicotine), suggesting a non-competitive inhibition. All symbols were normalized to the peak current induced by 100 µM nicotine, (averaged from 6 cells for nicotine, and 6 cells for nicotine plus 10 nM α-synuclein). In all recordings (**A**, **B** and **C**), the cells were held at a holding potential (V_H_) of −60 mV. In **B**, the double asterisk means *p*<0.01. Vertical bars indicate SEM.

To characterize the inhibitory effects on hα4β2-nAChR function induced by large oligomeric α-synuclein, we performed a series of studies using different concentrations of aggregated α-synuclein. Nicotine (3 µM, ∼EC50 concentration) was used as an agonist to activate hα4β2-nAChR expressed in SH-EP1 cells. α-Synuclein inhibited nicotine-induced whole-cell currents in a concentration-dependent manner (Fig. 4A and B; n = 6). Dose-dependent profiles of nicotine-induced whole-cell peak currents in the presence or absence of 10 nM aggregated α-synuclein (monomeric equivalent) showed a significant reduction in the maximal concentration of the nicotine-induced peak current but no change in nicotine EC_50_ values and Hill coefficients (2.7±0.6 and 1.0±0.08, for nicotine alone, *n* = 8; 3.6±0.8 and 1.1±0.07 for nicotine plus 10 nM α-synuclein, *n* = 8, *p*>0.05; t-test, Fig. 4C). These results suggest a non-competitive mechanism of α-synuclein–mediated inhibition.

### Effects of Large Oligomeric α-synuclein on Different nAChR Subtypes

**Figure 5:**
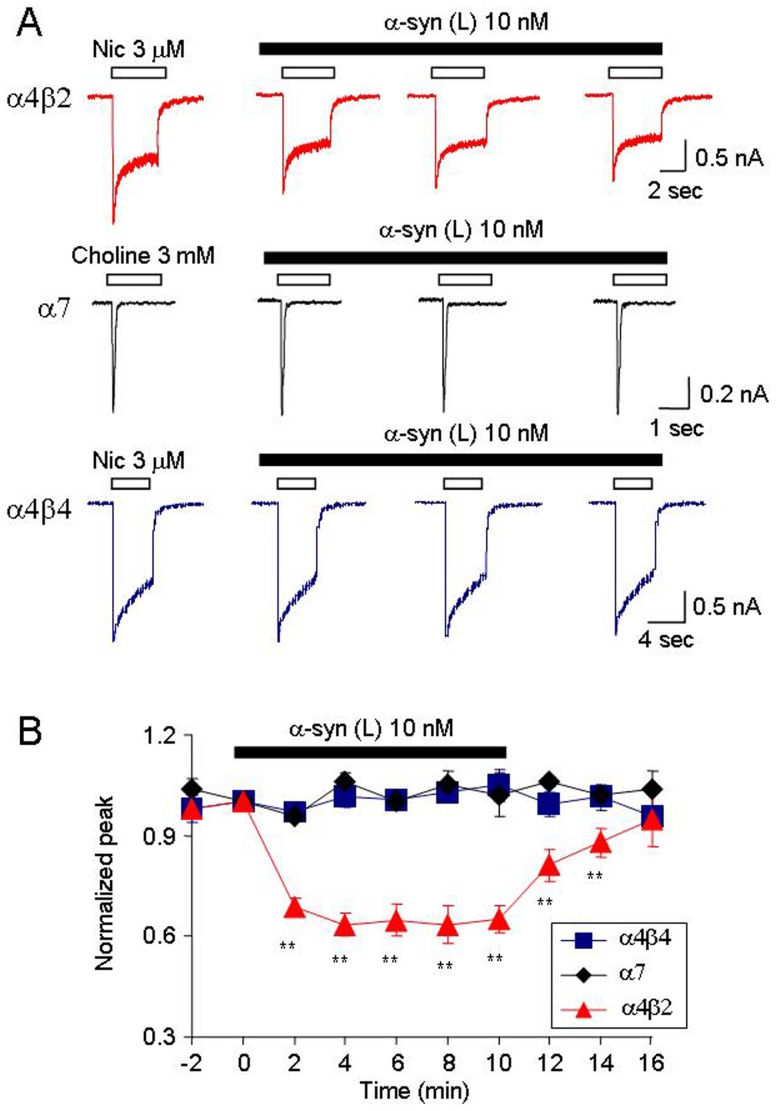
α-Synuclein inhibits hα4β2- but not α7- or α4β4- nAChR function. **A**. Representative whole-cell current traces comparing effects of 10 nM α-synuclein on α4β2-, α7-, and α4β4-nAChRs function. **B**. Summary of the effects of 10 nM α-synuclein on the peak currents for responses mediated by human α4β2-nAChRs (triangle), α7-nAChRs (diamond), or α4β4-nAChRs (square). The asterisk indicates *p*<0.05 and the double asterisk indicates *p*<0.01. Vertical bars indicate SEM.

Since large oligomeric α-synuclein inhibited hα4β2-nAChR currents, we studied if there was a differential effect of this α-synuclein aggregate species on different nAChR subtypes. We compared effects of the large oligomeric α-synuclein on hα4β2-, hα7- and hα4β4-nAChRs heterologously expressed in an SH-EP1 cell line. The hα4β2- and hα4β4-nAChRs were activated using 3 µM nicotine, while hα7-nAChR was activated using 3 mM choline. The results indicate that large oligomeric α-synuclein (at a pathophysiologically relevant concentration of 10 nM monomeric equivalent) [43] inhibited nicotinic peak current responses mediated by hα4β2-nAChRs, but not the current responses mediated by either hα7- or hα4β4-nAChRs (Fig. 5A). Statistical analysis showed that large oligomeric α-synuclein species reduced the peak amplitude of hα4β2-nAChR-mediated current to 62.9±3.7% (n = 6, *p*<0.01, multi-variate ANOVA); of hα7-nAChR-mediated current to 93.1±7.9% (n = 6, *p*>0.05, multi-variate ANOVA); and of hα4β4-nAChR-mediated current to 96.5±3.8% (n = 6, *p*>0.05, multi-variate ANOVA), respectively. These results indicate that the hα4β2-nAChRs are more sensitive to α-synuclein than hα7- and hα4β4-nAChRs.

### Mechanisms Involved in α-synuclein-induced Inhibition of hα4β2-nAChR Function

**Figure 6:**
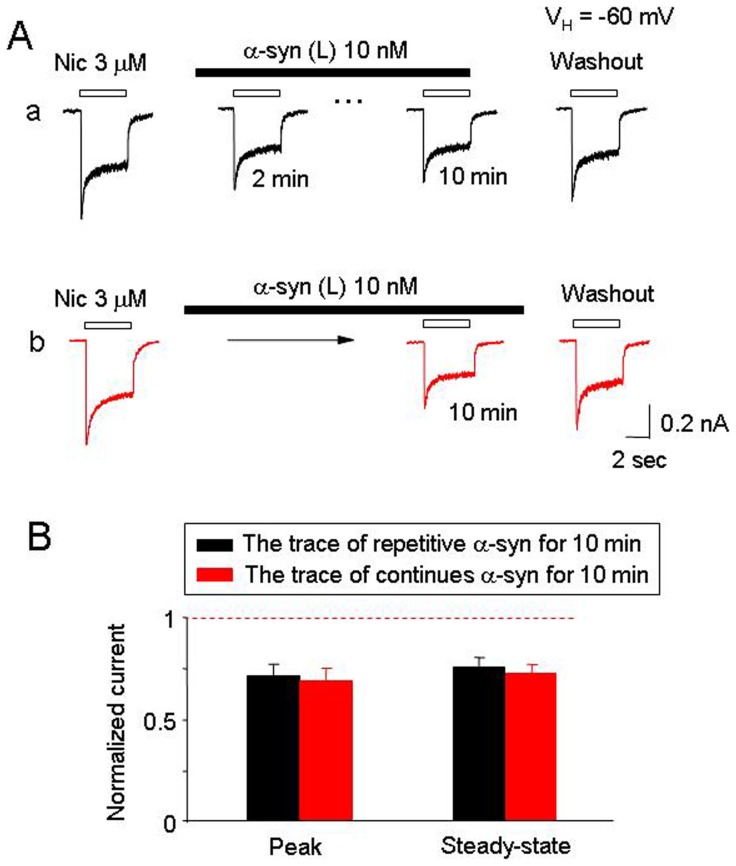
α-Synuclein is not an open-channel blocker to hα4β2-nAChR. **A**. Representative hα4β2-nAChR mediated whole-cell currents induced by repetitive applications nicotine (4 sec exposure at an interval of 2 min). The first response was recorded as controls. In **Aa**, nicotine exposures were repeated at 2-min intervals in the presence of 10 nM α-synuclein for 10 min, and subsequent response to nicotine was recorded after 6 min of washout of α-synuclein. In **Ab**, nicotine was applied at the end of the α-synuclein treatment (10 min), and a subsequent response to nicotine was recorded after 6 min of washout of α-synuclein. **B**. Bar graph summarizes replicated recordings of effects of 10 nM α-synuclein on nicotinic responses with and without repeated nicotine exposure. Data were collected from 8 cells in each group tested.

**Figure 7:**
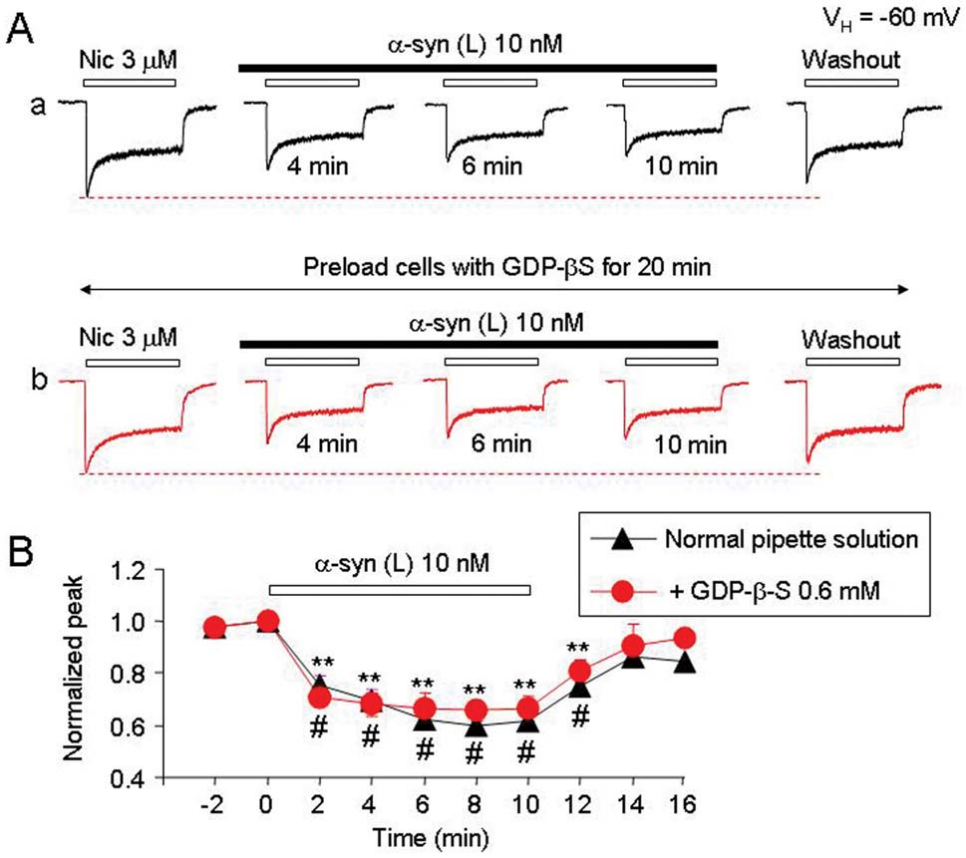
Inhibition of hα4β2-nAChRs by α-synuclein is not mediated through receptor turnover or internalization. A . Representative whole-cell current traces using typical tris-filled pipettes (**Aa**) and the tris-filled pipettes supplemented with 600 µM GDP-β-S (**Ab**). Initial responses to nicotine were measured after 20 min of the formation of conventional whole-cell recording (infusion of intracellular GDP-β-S into recorded cell). Sequence of drug applications are indicated as horizontal bars. **B**. Temporal patterns show the similar effects of 10 nM α-synuclein on nicotinic responses with (n = 8) and without (n = 8) GDP-β-S in the pipette solution. The double asterisks indicate *p*<0.01 compared before and after α-synuclein exposure, while # means *p*>0.05 compared between the pipette solution with (red) and without (black) GDP-β-S.

Since oligomeric α-synuclein selectively inhibits hα4β2-nAChRs function, we next determined whether α-synuclein was functioning through either of two different potential mechanisms: 1) via a mechanism of open channel block, or 2) via induction of hα4β2-nAChR internalization. To test whether α-synuclein-induced inhibition operated through the open channel block mechanism, we repeatedly applied 3 µM nicotine in the continuous presence of 10 nM large oligomeric α-synuclein. Repeated application of nicotine (2 min interval) in the presence of 10 nM α-synuclein for 10 min led to a reduction of nAChR response (Fig. 6Aa), while continuous application of α-synuclein for 10 min without repetitive exposure to nicotine led to similar inhibition (Fig. 6Ab). The peak component of repetitive nicotine-induced current was reduced to 70.8±5.8% (Fig. 6Aa, n = 8, *p*<0.01, t-test) and by non-repetitive challenges of nicotine, the peak current was reduced to 67.5±6.9% (Fig. 6Ab, n = 8, *p*<0.01, t-test). No statistical significance in the nicotine-induced currents was observed between the two protocols described above (Fig.6B, n = 8, p>0.05, t-test). Thus, 10 nM large oligomeric α-synuclein did not show clear signs of use-dependent inhibition of hα4β2-nAChRs (Fig. 6A and B). The absence of a use-dependence feature suggests that non-competitive inhibition of hα4β2-nAChR function by α-synuclein is not mediated through an open channel block. To investigate whether or not α-synuclein-induced hα4β2-nAChR internalization is involved, patched cells were preloaded with GDP-β-S (600 µM) for 20 min, which has previously been reported to prevent α-amino-3-hydroxy-5-methyl-4-isoxazolepropionate receptor endocytosis [44]. GDP-β-S treatment neither prevented α-synuclein-mediated inhibition nor improved hα4β2-nAChR functional recovery from washout of α-synuclein (Fig. 7, A and B). These data suggest that inhibition of hα4β2-nAChR function by α-synuclein is also not mediated by hα4β2-nAChR internalization through a process affecting α-amino-3-hydroxy-5-methyl-4-isoxazolepropionate receptors.

## Discussion

In the present study, we show that pathologically-relevant levels (10 nM monomeric equivalent) [43] of aggregated α-synuclein inhibit human α4β2-nAChR function. We find that larger oligomeric α-synuclein aggregate species (>4 nm) but not monomeric, fibrillar or smaller α-synuclein aggregates (2–4 nm) are responsible for this partial inhibitory effect on α4β2-nAChRs. The partial inhibitory effect of α-synuclein on hα4β2-nAChRs exhibits a mechanism that is dose-dependent, non-competitive, non use-dependent and non-internalization based. Interestingly, the α-synuclein-induced inhibition occurs more profoundly in α4β2-nAChRs than in other nAChR subtypes such as α7- or α4β4-nAChRs, indicating subtype selectivity.

### Distinguish Inhibitory Effects on nAChRs by Different Forms of α-synuclein

Misfolding and aggregation of α-synuclein has been implicated in the pathogenesis of numerous neurodegenerative diseases, particularly in PD [4], [45]. Different oligomeric α-synuclein species can be generated as intermediate species during the transition from monomeric to fibrillar aggregates [46], [47]. Accumulating evidence indicates that oligomeric α-synuclein is the most toxic species responsible for neurodegeneration and neuronal loss in PD [48], [49], [50]. nAChRs have been linked to pathogenesis of PD and recent evidence suggests possible roles for nAChRs as potential targets for α-synuclein-induced neurotoxicity manifest as cholinergic hypofunction in PD [26], [27], [33]. However, whether or not α-synuclein directly modulates nAChR function, especially whether specific aggregated morphologies of α-synuclein interact with different subtypes of nAChRs has not been examined previously. Several studies have shown that different aggregated α-synuclein forms added extracellularly to the culture medium can have different cytotoxic effects [15], [16], [17], [18], [20], [21]. Here we show that oligomeric but not monomeric or fibrillar α-synuclein directly inhibits the function of hα4β2-nAChRs. To further study the effects of oligomeric α-synuclein on hα4β2-nAChRs, we determined whether there are any differences in the inhibitory effects of different aggregate forms of α-synuclein toward hα4β2-nAChRs. Size exclusion chromatography was used to separate several distinct aggregate species of α-synuclein. We show that a large oligomeric α-synuclein aggregate species (predominantly, >4 nm, 99.6%), but not small aggregate species (2–4 nm, 87.4%) significantly inhibited hα4β2-nAChRs function, indicating that morphologically distinct forms of α-synuclein result in different nAChRs inhibition potency. These studies support the hypothesis that aggregated α-synuclein, particularly oligomeric species, may target hα4β2-nAChRs expressing dopaminergic neurons during the pathogenesis of PD and may account for the loss of cholinergic input to dopaminergic neurons [30], [51].

### Possible Mechanisms of α-synuclein-mediated Inhibition of h*α*4*β*2-nAChR Function

These results clearly demonstrate that oligomeric α-synuclein selectively and partially inhibits hα4β2-nAChR function. The finding of non-competitive antagonism of hα4β2-nAChRs by oligomeric α-synuclein suggests that the large oligomeric α-synuclein species acts as a non-competitive antagonist of hα4β2-nAChRs under our experimental conditions. Our results also demonstrate that oligomeric α-synuclein, at concentrations from 1 nM to 1 µM, failed to directly induce whole-cell current responses from cells expressing hα4β2- or hα7-nAChRs or from untransfected cells (data not shown). These results indicate that α-synuclein in our hands does not have properties of a nAChR agonist.

Additionally, our data show no use-dependence of large oligomeric α-synuclein-induced inhibition on hα4β2-nAChR function, suggesting that the inhibitory effects are not mediated by open channel block, although the persistence in functional block after washout of fluid phase α-synuclein suggests a “lingering” effect of the aggregates. We found that pretreatment with large oligomeric α-synuclein is necessary to induce convincing inhibition of hα4β2-nAChR-mediated whole-cell currents. However, the nature of this inhibition is still unknown. One potential explanation is that there is an aggregated α-synuclein-driven long-lasting closed conformation of nAChRs. This idea is supported by the present observation that long exposure times (10 min) to large oligomeric α-synuclein aggregates leads to persistent loss of hα4β2-nAChR function, and any loss of nAChR function does not appear to be mediated via α-synuclein-induced hα4β2-nAChR internalization. This conclusion is based on the observation that GDP-β-S (600 µM) fails to prevent the loss of hα4β2-nAChR function induced by α-synuclein pre-incubation. On the other hand, the inhibition of hα4β2-nAChR function by α-synuclein is partial, non-competitive, and reversible, which is similar to amyloid-induced inhibition on hα4β2-nAChR [5]. In fact, pre-treatment with oligomeric amyloid 1–42 (1 nM) for 10 min prevented α-synuclein-induced inhibition in hα4β2-nAChR-mediated currents (Figure S1), suggesting a mechanism reminiscent of a negative allosteric inhibitor. The relatively large size of the inhibitory species may be indicative of a physical interaction partially inhibiting hα4β2-nAChR function but not totally blocking it.

In addition, it is also interesting that α-synuclein selectively inhibits hα4β2-nAChR subtype rather than hα7- or hα4β4-nAChR subtypes. Although it has been reported that α7 nAChRs, shown to be unaffected in the present report by α-synuclein, are upregulated in PD [31], there are experimental differences between our studies and that of Guan et al. that make direct comparison difficult. For example, we examined direct effects of acute exposure of α-synuclein on nAChR function using transfected nAChRs in cell lines, while Guan et al. reported the nAChR subunit expression and binding using PD brain tissue [31]. The potential effects of α-synuclein-induced inhibition remain to be examined on other nAChR subtypes, such as hα6β2-, hα6β2β3- and hα3β4-nAChR, which may serve as a potential target for PD therapeutics as well. These α6-containing nAChR subtypes may be important since they show significant declines in PD animal models. However, due to difficulties in stably expressing these heterologous α6-containing receptors in SH-EP-1 cell lines, we were not able to test the effects of α-synuclein on these subtypes of nAChRs here.

### Pathological Relevance of *α*4*β*2-nAChR Dysfunction and PD

Neuronal nicotinic receptors that bind radiolabeled nicotine with the highest affinity contain α4 subunits (α4*-nAChR) [52], [53]. Immunoassays have shown that the predominant, naturally expressed form of the α4*-nAChR in the vertebrate brain contains α4 and β2 subunits (α4β2-nAChR) [54], [55]. Evidence indicates that a consistent, significant loss of α4*-nAChRs has been observed at autopsy in PD brain [31], [56], [57], [58]. The major pathological features of PD are α-synuclein protein deposition, lewy body formation, and a severe dopaminergic deficit [32]. It has been shown that the α-synuclein protein is a major constituent of lewy bodies, a neuropathologic hallmark of PD [32]. However, links between soluble α-synuclein accumulation and cholinergic dysfunction remain unclear. The present study characterized the aggregated morphologies of α-synuclein by size exclusion chromatography and AFM. This enabled us to distinguish different aggregated α-synuclein species. Oligomeric α-synuclein, particularly the larger oligomeric α-synuclein aggregates studied here, selectively inhibits hα4β2-nAChR function in a dose-dependent and non-competitive manner, providing the basis for a new hypothesis that α-synuclein can directly modulate hα4β2-nAChR function, which in turn may contribute to cholinergic signaling deficits in PD.

Although a partial inhibitory effect of α-synuclein on hα4β2-nAChR function was observed at pathophysiology relevant concentrations, further investigation is needed to determine whether such an effect will be large enough to be clinically relevant. It is also noteworthy that α7-nAChR binding sites are increased in PD brain tissues, suggesting that α7-nAChR might be affected by α-synuclein during PD pathogenesis. However, under our conditions, direct acute exposure of α-synuclein fails to affect hα7-nAChR function. One possible interpretation is that there may be other confounders during chronic α-synuclein accumulation to affect α7-nAChR expression *in vivo*, which cannot be mimicked by acute, *in vitro* experiments. Our perspective on a primary role for hα4β2-nAChRs in low concentration effects of α-synuclein complements other findings that the modulation of nAChR function by α-synuclein could be pathologically relevant [27], [32], [33].

### Conclusion

Collectively, our findings demonstrate for the first time that pathologically-relevant concentrations of aggregated oligomeric α-synuclein directly inhibit neuronal human α4β2-nAChR function. We find that large oligomeric α-synuclein aggregates (>4 nm), but not monomeric or fibrillar α-synuclein, selectively inhibit hα4β2-nAChR function starting at 10 nM (monomeric equivalent). Specifically, we show that predominantly larger oligomeric α-synuclein aggregates (>4 nm) but not smaller species (<4 nm) potently inhibit hα4β2-nAChRs mediated whole-cell currents. Furthermore, we elucidate pharmacological mechanisms of α-synuclein–induced inhibition, which includes dose-dependent, non-competitive, non-use-dependent manners, and this inhibition is not mediated through nAChR internalization. Finally, we demonstrate that the functional inhibition by α-synuclein exhibits nAChR subunit selectivity, occurring more profoundly in α4β2-nAChRs than in other nAChR subtypes such as α7- or α4β4-nAChRs. Our findings, along with previous reports on the roles of α6β2*-nAChRs in PD pathogenesis [33], suggests that nAChRs are sensitive targets for α-synuclein toxicity. The α4β2-nAChRs are sensitive to morphologically specific and pathologically relevant concentrations of α-synuclein, suggesting that novel strategies for PD therapy could involve amelioration of specific aggregated α-synuclein-induced α4β2-nAChR functional deficits and/or perhaps preservation of α4β2-nAChR function.

## Supporting Information

Figure S1 Effects of pretreatment of oligomeric amyloid (Aβ1-42) on α-synuclein-induced inhibition of human α4β2-nAChRs heterologously expressed in SH-EP1 cell line. We found that after 10 min pre-treatment with 1 nM oligomeric Aβ1-42, 3 µM nicotine (around EC50 concentration)-induced inward current was reduced (Figure S1A, blue trace). Thereafter, we immediately added 10 nM α-synuclein (in the continuous presence of 1 nM Aβ1-42) for 10 min, and then tested nicotinic response. However, we did not observe further reduction of nicotine-induced inward current (Figure S1A, red trace). Statistic analysis showed that Aβ1-42 pre-treatment significantly reduced both peak and steady-state components of nicotine-induced-whole-cell current (Figure S1B, n = 6, *p*<0.01), while in the presence of Aβ1-42, α-synuclein failed to further reduce this current response (*p*>0.05 between Aβ1-42 and α-synuclein treated group), indicated as no significance (NS) in the figure. These results suggest that both oligomeric molecules of Aβ1-42 and α-synuclein likely bind to a common negative allosteric site to reduce human α4β2-nAChR function.
(DOC)
