# Supplementary material for: Human α4β2 Nicotinic Acetylcholine Receptor as a Novel Target of Oligomeric α-Synuclein
Source: PLoS One. 2013 Feb 20;8(2):e55886. doi: 10.1371/journal.pone.0055886 (PMC3577813; doi:10.1371/journal.pone.0055886)
Supplement: Figure S1 — Effects of pretreatment of oligomeric amyloid (Aβ1-42) on α-synuclein-induced inhibition of human α4β2-nAChRs heterologously expressed in SH-EP1 cell line. We found that after 10 min pre-treatment with 1 nM oligomeric Aβ1-42, 3 µM nicotine (around EC50 concentration)-induced inward current was reduced (Figure S1A, blue trace). Thereafter, we immediately added 10 nM α-synuclein (in the continuous presence of 1 nM Aβ1-42) for 10 min, and then tested nicotinic response. However, we did not observe further reduction of nicotine-induced inward current (Figure S1A, red trace). Statistic analysis showed that Aβ1-42 pre-treatment significantly reduced both peak and steady-state components of nicotine-induced-whole-cell current (Figure S1B, n = 6, p<0.01), while in the presence of Aβ1-42, α-synuclein failed to further reduce this current response (p>0.05 between Aβ1-42 and α-synuclein treated group), indicated as no significance (NS) in the figure. These results suggest that both oligomeric molecules of Aβ1-42 and α-synuclein likely bind to a common negative allosteric site to reduce human α4β2-nAChR function. (DOC) [file pone.0055886.s001.doc]

Figure S1. Effects of pretreatment of oligomeric amyloid (Aβ1-42) on α-synuclein-induced inhibition of human α4β2-nAChRs heterologously expressed in SH-EP1 cell line. We found that after 10 min pre-treatment with 1 nM oligomeric Aβ1-42, 3 μM nicotine (around EC50 concentration)-induced inward current was reduced (Figure S1A, blue trace). Thereafter, we immediately added 10 nM α-synuclein (in the continuous presence of 1 nM Aβ1-42) for 10 min, and then tested nicotinic response. However, we did not observe further reduction of nicotine-induced inward current (Figure S1A, red trace). Statistic analysis showed that Aβ1-42 pre-treatment significantly reduced both peak and steady-state components of nicotine-induced-whole-cell current (Figure S1B, n=6, *p*<0.01), while in the presence of Aβ1-42, α-synuclein failed to further reduce this current response (*p*>0.05 between Aβ1-42 and α-synuclein treated group), indicated as no significance (NS) in the figure. These results suggest that both oligomeric molecules of Aβ1-42 and α-synuclein likely bind to a common negative allosteric site to reduce human α4β2-nAChR function.


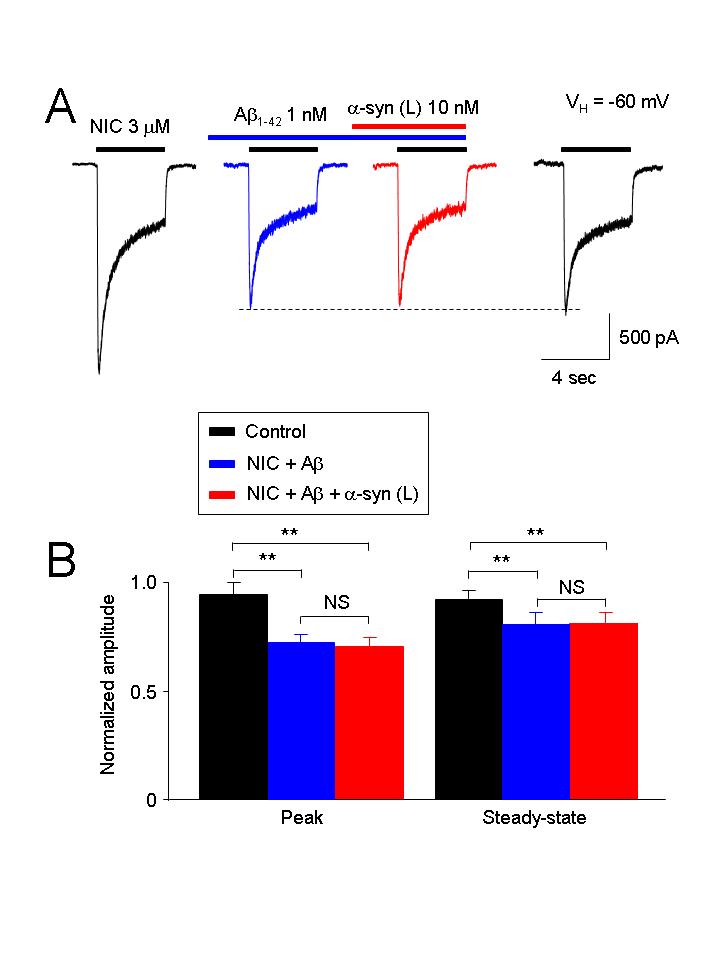


Figure S1. Pretreatment with Aβ1-42 prevents inhibitory effects of large oligomeric α-synuclein on α4β2-nAChRs function. **A.** Representative typical whole-cell current traces, in which the effects of 1 nM Aβ1-42 (10 min pre-treatment) on 10 nM α-synuclein (10 min pre-treatment)-induced reduction of α4β2-nAChR function. Results showed after 10 min pre-treatment with 1 nM Aβ1-42, 10 nM α-synuclein (10 min pre-treatment in the presence of 1 nM Aβ1-42) failed to further reduce nicotine-induced whole-cell currents. **B.** Summarized observations of effects of 10 nM α-synuclein (10 min) on the peak or steady-state currents for responses mediated by human α4β2-nAChRs after pretreatment with 1 nM Aβ1-42 (10 min). Each column was averaged from 6 cells tested. The double asterisk means p < 0.01. Vertical bars indicate SEM.
